# Thrombose de la veine dorsale profonde de la verge revelant une maladie de Behcet

**DOI:** 10.11604/pamj.2016.24.17.9309

**Published:** 2016-05-06

**Authors:** Ali Beddouche, Hicham Ouaziz, Sinane Zougaghi, Abdelilah Alaoui, Hamza Dergamoun, Hachem El Sayegh, Ali Iken, Lounis Benslimane, Yassine Nouini

**Affiliations:** 1 Service d'Urologie A, Hôpital Ibn Sina, CHU Rabat, Maroc

**Keywords:** Thrombose, veine dorsale, verge, maladie de Behçet, Thrombosis, dorsal vein, penis, Behcet's disease

## Abstract

La thrombose de la veine dorsale profonde de la verge (TVDPV) est une urgence rare et mal connue en urologie. Elle nécessite une prise en charge précoce symptomatique et étiologique afin de préserver la fonction érectile et d’éviter les récidives. Nous rapportant à travers notre observation un cas de thrombose veineuse dorsale de la verge révélée par un priapisme spontané non résolutif, et confirmé par un écho-doppler pénien. Apres prise en charge du priapisme et de la TVDPV, l'enquête étiologique a révélé une maladie de Behçet dont le diagnostic a été retenu sur l'association d'un critère majeur qui est l'aphtose buccale, et de 3 critères mineurs que sont: l'aphtose génitale, l'atteinte oculaire, et un test pathergique cutané positif à 24h. Un traitement étiologique a été instauré avec bonne évolution clinique, et conservation de la fonction érectile.

## Abstract (en)

Deep dorsal penile vein thrombosis (DDPVT)is a rare and little known urologic emergency. It requires an early etiological and symptomatic approach to preserve erectile function and prevent recurrences. This study reports a case of dorsal penile vein thrombosis revealed by spontaneous priapism that didn't resolve adequately and confirmed by penile Doppler ultrasound. After management of priapism and DDPVT, the etiological investigation revealed Behcet's disease whose diagnosis was based on the association of a major criteria, such as oral aphthous ulcers with 3 minor criteria such as: genital aphthous ulcers, ocular involvement, and a positive skin pathergy test within 24h. The patient underwent etiological treatment with good clinical evolution and preservation of erectile function.

## Introduction

La thrombose de la veine dorsale profonde de la verge (TVDPV) est une pathologie rare et mal connue en urologie. Il s'agit d'une urgence diagnostique et thérapeutique, nécessitant une prise en charge rapide symptomatique et étiologique. Sur le plan physiopathologique, la TVDPV bloque le retour veineux provoquant une congestion des corps caverneux avec compression des artères intra-caverneuses pouvant aboutir à une ischémie pénienne et compromettre la fonction érectile [1]. A travers notre observation nous rapportons un cas exceptionnel de thrombose de la veine dorsale de la verge révélant une maladie de Behçet.

## Patient et observation

**Figure 1:**
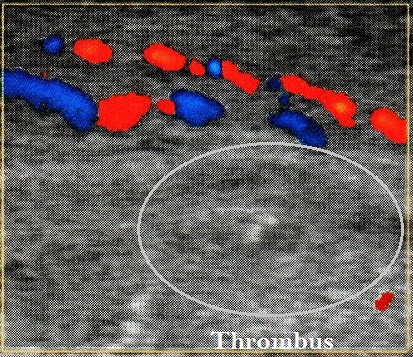
Echo-doppler pénien: thrombus échogène au sein d'une veine distendue et incompressible

**Figure 2:**
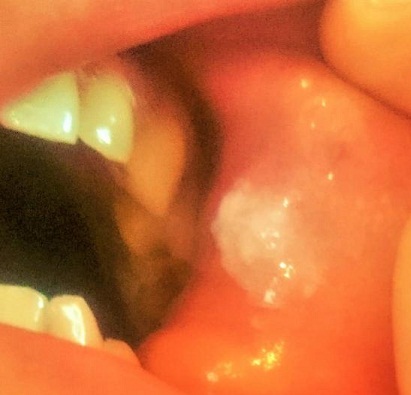
Aphte de la face interne de la joue

**Figure 3:**
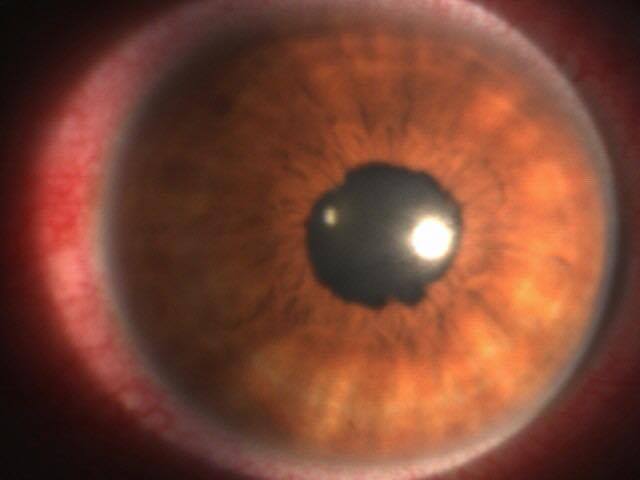
Uvéite antérieure lors de l'examen à la lampe à fente

**Figure 4:**
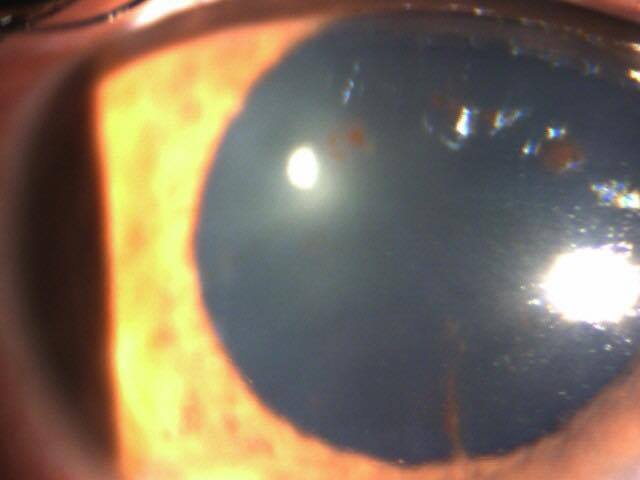
Uvéite antérieure lors de l'examen à la lampe à fente après dilatation

Nous rapportons le cas d'un patient âgé de 54 ans, sans antécédents pathologiques particuliers, consultant pour un priapisme spontané, douloureux et non résolutif, évoluant depuis plus de 6 heures. Un écho-doppler pénien réalisé en urgence a révélé une thrombose de la veine dorsale de la verge (Figure 1). Le patient a bénéficié initialement d'injections intra-caverneuses d'alpha- stimulants (Ephédrine), d'une ponction aspiration trans-glandulaire, ainsi qu'un traitement anticoagulant à base d'héparine de bas poids moléculaire relayé par les anti-vitamines K. Après détumescence satisfaisante et amélioration des symptômes, la reprise de l'interrogatoire a révélé la survenue récurrente d'aphtoses buccales, de polyarthralgies asymétriques intéressant les grosses articulations, ainsi qu'une baisse récente de l'acuité visuelle. L'examen clinique a retrouvé un aphte buccal (Figure 2), des lésions cicatricielles d'ulcérations génitales, ainsi qu'une uvéite antérieure lors de l'examen à la lampe a fente (Figure 3, Figure 4). Le bilan biologique a révélé un syndrome inflammatoire isolé, sans trouble d'hémostase, ou hémopathie associée. Le diagnostic de maladie de Behcet a été retenu selon les nouveaux critères de l'American College of Rhumatology de 2007, sur l'association d'un critère majeur qui est l'aphtose buccale, et de 3 critères mineurs que sont: l'aphtose génitale, l'atteinte oculaire, et un test pathergique cutané positif à 24h. parallèlement aux AVK, le patient a été mis sous colchicine pour l'aphtose buccale, corticoïdes et mydriatique pour l'uvéite antérieure, ainsi qu'un traitement immunosuppresseur à base d'Azathioprine. L’évolution a été marquées par une amélioration des symptômes, avec conservation de la fonction érectile.

## Discussion

La thrombose de la veine dorsale de la verge est une pathologie rare en urologie, dont la symptomatologie est polymorphe, pouvant aller de la simple douleur à type de lourdeur, à l’œdème jusqu'au priapisme [1].

Le diagnostic repose principalement sur l’écho-doppler veineux par la mise en évidence d'un thrombus échogène au sein d'une veine distendue et incompressible, avec absence de flux dans la veine dorsale profonde de la verge en doppler couleur [2].

La prise en charge repose sur le traitement précoce du priapisme s'il existe, de la thrombose (traitement anticoagulant à base d'héparine de bas poids moléculaire relayé par les anti-vitamines K), ainsi que l’étiologie afin de préserver la fonction érectile et d’éviter les récidives [3, 4]. Les étiologies de la thrombose veineuse profonde de la verge sont celles de toute thrombose veineuse: les maladies de système (dont la maladie de Behçet), les hémophilies, les hémopathies, les cancers, la chirurgie et les traumatismes [5, 6].

Décrite pour la première fois en 1937 par le professeur Turque Hulusi Behçet en 1937 [7], la maladie qui porte son nom est une vascularite multisystémique caractérisée par des aphtes buccaux récidivants, des ulcères génitaux, une atteinte inflammatoire oculaire, des lésions cutanées et une atteinte fréquente des articulations. Le système nerveux central, le tractus gastro-intestinal et les vaisseaux sont moins fréquemment touchés, mais leur atteinte peut donner lieu à des complications vitales.

Les atteintes vasculaires appelées aussi angio-behcet touchent le plus souvent l'adulte jeune de sexe masculin et surviennent dans 46% des cas. Il s'agit le plus souvent de thromboses veineuses se localisant essentiellement dans les veines périphériques. Les atteintes artérielles sont plus rares et surviennent dans 16% des cas [8].

Le diagnostic de La maladie de Behçet est essentiellement clinique, et fait appel aux critères internationaux publiés en 1990 proposés par l'international study group for Behçet's disease (Tableau 1) [9], et révisés en 2007 pour plus de sensibilité et de spécificité [10]. Le diagnostic est retenu devant l'association d'un critère majeur, qui est l'aphtose buccale (au moins 3 x/année), et au moins deux critères mineurs parmi lesquels on trouve les ulcérations génitales récurrentes, les lésions oculaires, les lésions cutanées et le test pathergique positif.

**Tableau 1:** Critères de classification de la maladie de Behçet, proposés par l'International Study Group for Behçet's Disease [9]

**Aphtose buccale récidivante**	3 types: majeur, mineur, herpétiforme
	3 types: majeur, mineur, herpétiforme
	Observé par un médecin ou le patient
**+ au moins 2 des critères suivants:**	
**Ulcérations génitales récidivantes ou lésions cicatricielles**	Observées par un médecin ou le patient
**Lésions oculaires**	Uvéite antérieure, uvéite postérieure, hyalite à la lampe à fente.
	Vasculite rétinienne observé par un ophtalmologue.
**Lésions cutanées**	
	Érythème noueux, pseudofolliculite, lésions papulo-pustuleuses.
	Nodules acnéiformes observés par un médecin en dehors de l'adolescence ou d'un traitement corticoïde.
**Test pathergique cutané positif**	Lu par un médecin après 24-48h.

Le traitement de la maladie de Behçet est symptomatique et dépend essentiellement de ses manifestations cliniques. Un traitement immunosuppresseur peut être envisagé devant toute aggravation ou récurrence de ses symptômes malgré un traitement adapté.

## Conclusion

La thrombose de la veine dorsale profonde de la verge est une urgence urologique. Le traitement doit être instauré rapidement sinon le pronostic sexuel peut en être compromis. L'association possible à d'autres pathologies doit inciter à réaliser un bilan étiologique en fonction du contexte clinique, et instaurer un traitement adapté afin d ’éviter les récidives.
